# Deceleration of fetal growth rate as alternative predictor for childhood outcomes: a birth cohort study

**DOI:** 10.1186/s12884-019-2358-8

**Published:** 2019-06-27

**Authors:** Zoe A. Broere-Brown, Sarah Schalekamp-Timmermans, Vincent W. V. Jaddoe, Eric A. P. Steegers

**Affiliations:** 1 Department of Obstetrics and Gynecology, Erasmus MC - University Medical Center, room Na 2918, PO Box 2040, 3000 CA Rotterdam, The Netherlands; 2 Department of Epidemiology, Erasmus MC – University Medical Center, Rotterdam, The Netherlands; 3 Department of Pediatrics, Erasmus MC – University Medical Center, Rotterdam, The Netherlands

**Keywords:** Birth weight, Small for gestational age, Fetal growth restriction

## Abstract

**Background:**

Small for gestational age (SGA) is frequently used to define fetal growth restriction (FGR). However, FGR describes a slowdown in fetal growth and is not synonymous with SGA, which may introduce misclassification. We investigated the effect of both on delivery and childhood outcomes.

**Methods:**

From a prospective population-based cohort study we included 7959 live singleton births with data available on second trimester estimated fetal weight (EFW) and birth weight. We used a decrease in growth of > 40 percentiles between second trimester EFW and birthweight to define a deceleration in growth. SGA was defined as birthweight <p5.

**Results:**

Deceleration of growth occurred in 27,2% in SGA neonates and in 10,3% of neonates with an appropriate for gestational age (AGA) birthweight. Of all fetuses with decelerated growth, 90% was born AGA. SGA neonates were more often delivered by instrumental delivery or cesarean section and admitted to NICU. Both decelerated growth and SGA were associated with accelerated growth at 2 years, a smaller aortic diameter and lower left ventricular mass at 6 years.

**Conclusions:**

Both decelerated growth and SGA are associated with unfavorable clinical outcomes in childhood. In addition to SGA, neonates with deceleration of growth should be considered a high-risk group.

**Electronic supplementary material:**

The online version of this article (10.1186/s12884-019-2358-8) contains supplementary material, which is available to authorized users.

## Background

Fetal growth restriction (FGR) is considered a severe complication of pregnancy associated with substantial perinatal morbidity and mortality and contributing to disease in adulthood [1, 2]. The Development and Origins of Health and Disease theory (DOHaD) states that in case of adverse fetal exposure, the unborn fetus can modify its own development such that it will be prepared for survival in an environment in which resources are likely to be short. Although these adaptations may be beneficial for short term survival, they may have adverse consequences at delivery or in later life [3]. FGR is difficult to assess as the biological growth potential of the fetus can, at best, be estimated and not directly measured. Therefore, in scientific research FGR is frequently classified as a neonate born small for gestational age (SGA). Yet, birth weight and thus one single measurement can only indicate size. Growth however is dynamic and can be measured only in sequential measurements. Therefore FGR is not synonymous with SGA. FGR fetuses may experience a failure to reach their biological growth potential because of a pathological slow-down (decelerating growth curve) in the fetal growth pace. However, there is a lack of a uniform definition of decelerated growth and no golden standard exists. This is highly warranted since it is estimated that approximately 50–70% of the SGA fetuses are constitutionally small with normal perinatal outcomes [4, 5]. Growth velocity represents the rate of fetal growth in a specific time interval and may have more clinical utility to distinguish normal from pathological fetal growth and hence to identify fetal growth abnormalities.

In this study we assessed decelerating growth based on the fetus individual growth curve, independent of birth weight. We compared this method to small for gestation age (SGA) to determine whether growth velocity, independent of SGA, affects delivery outcomes, accelerated growth in infancy and cardiovascular outcomes at the age of 6 years.

## Methods

This paper is a subset of the thesis of Dr. Broere-Brown entitled Fetal sex dependency in pregnancy, fetal and maternal outcomes (https://www.generationr.nl/wp-content/uploads/2017/10/PROEFSCHRIFT-ZA-BROERE-BROWN.pdf).

### Study design

**Fig. 1:**
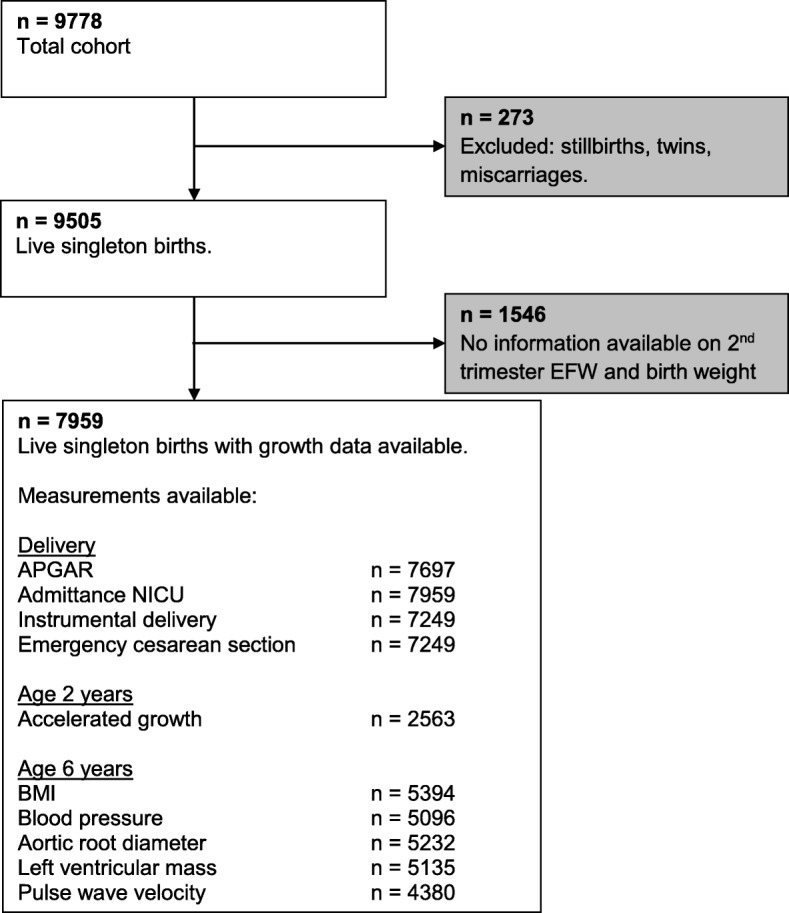
Flowchart

The study was embedded in The Generation R Study, a population-based prospective cohort study from early pregnancy onwards [6]. All mothers with an expected delivery date between April 2002 and January 2006 were eligible. Response at baseline was 61%. The Medical Ethics Committee (MEC) of the Erasmus Medical Center Rotterdam, The Netherlands approved the study in 2001 (MEC 198.782/2001/31). Written informed consent was obtained from all mothers. For the present study we included pregnancies with a live born singleton birth with a known second trimester estimated fetal weight (EFW) and birth weight (*N* = 7959) (Fig. 1).

### Fetal growth

To assess estimated fetal weight (EFW) ultrasound examinations were performed in the second trimester of pregnancy (median 20.5 weeks of gestation, 90% range 18.9–22.9). Fetal biometry (head circumference [HC], abdominal circumference [AC} and femur length [FL]) was measured trans abdominally. EFW was calculated using the formula of Hadlock with parameters AC, HC and FL (in cm): EFW = 10**(1.326–0.00326*AC*FL + 0.0107*HC + 0.0438*AC + 0.158*FL [7]. Ultrasound examinations were performed using Aloka® model SSD-1700 (Tokyo, Japan) or the ATL-Philips® Model HDI 5000 (Seattle, WA, USA). SGA was defined as a gestational and fetal sex adjusted birth weight under the fifth percentile (≤ 1.78 SD).

Both estimated fetal weight in the second trimester of pregnancy and birth weight were presented in a gestational age adjusted percentile. Since there is no definition of how much a growth curve needs to decelerate before it can be designated as a decelerating growth curve we used five cut-offs to define decelerated growth; a decrease in growth of at least 30, 35, 40, 45 or 50 percentiles respectively between the second trimester and birth. This approach has been suggested in previous studies and aims to approach a slowdown in fetal growth. This slowdown is independent of the actual birth weight. For example a fetus with an EFW of p85 in the second trimester ending with a birth weight of p35 has a growth deceleration of 50 percentiles but does not meet the definition of SGA. However a fetus with an EFW of p55 and a growth deceleration of 50 percentiles ends with a birth weight of p5, which does meet the definition of SGA. We did not explore a decrease in growth of less than 30 percentiles since these deviations in growth could well be the results of measurement errors. The results using the cut-off of 40 percentiles are presented, results on the other cut-off values can be found in the Supplementary materials. Choosing cut-offs to define decelerated growth may lead to misclassification. The initial estimated fetal weight in the second trimester of pregnancy should be above the 40th percentile. Otherwise the fetus is not able to deviate from its growth curve with more than 40 percentiles. Therefore, in additional analyses we classified all fetuses with an EFW in the second trimester under the 40th percentile and with a maximum decrease of growth (birth weight under the first percentile) as decelerated growth (*n* = 85).

### Delivery outcomes

APGAR score at five minutes and delivery mode (spontaneous versus instrumental or emergency cesarean section) were obtained from standardized delivery registrations of midwives and obstetricians. An APGAR score below seven after five minutes was considered low [8, 9]. Information concerning admittance to the neonatal intensive care unit (NICU) was obtained using hospital and national registries.

### Infant growth

Well-trained staff in the Community Health Centers obtained postnatal growth characteristics according to standard schedule and procedures at the age of 24 months. Standard deviation scores for childhood weight were obtained with Dutch growth references charts (Growth Analyzer 3.0; Dutch Growth Research Foundation, Rotterdam, the Netherlands). Postnatal accelerated growth was defined as an increase between birth and 2 years of age in their position on the age-specific weight distribution by at least 0.67 SDS, representing the width of each percentile band on a standard growth chart [10, 11].

### Childhood cardiovascular outcomes

We invited all children to a dedicated research facility in the Erasmus University Medical Center, Sophia Children’s Hospital for detailed measurements at the age of 6 years (mean 6.2 ± 0.5) [6]. We measured height and weight and calculated body mass index (BMI). Systolic (SBP) and diastolic blood pressure (DBP) was measured at the right brachial artery by using the validated automatic sphygmomanometer Datascope Accutor Plus (Paramus, NJ, USA) [12]. We selected a cuff with a width approximately 40% of the arm circumference and long enough to cover 90% of the arm circumference.

Carotid-femoral pulse wave velocity (PWV) was assessed by using the automatic Complior SP device (Artech Medical, Pantin, France) with participants in supine position. The distance between the recording sites at the carotid (proximal) and femoral (distal) artery was measured over the surface of the body to the nearest centimeter. Through piezoelectric sensors placed on the skin, the device collected signals to assess the time delay between the upstroke of carotid and femoral waveforms. Carotid-femoral pulse wave velocity (PWV) was calculated as the ratio of the distance traveled by the pulse wave and the time delay between the waveforms, as expressed in meters per second [13, 14]. To cover a complete respiratory cycle, the mean of at least 10 consecutive pressure waveforms was used in the analyses. PWV can be measured reliably with good reproducibility in large pediatric population-based cohorts [14, 15].

Two-dimensional M-mode echocardiographic measurements were performed using the ATL-Philips Model HDI 5000 or the Logiq E9 (GE Medical Systems, Wauwatosa, Wisconsin, USA) devises. Echocardiography was used to measure the aortic root diameter (AOD), interventricular septum thickness in diastole (IVSTD), left ventricular internal diameter in diastole (LVIDD) and the left ventricular posterior wall thickness in diastole (LVPWTD) using methods recommended by the American Society of Echocardiography [16]. Left ventricular mass (LVM) was calculated using the formula derived by Devereux et al.: LVM = 0.80 × 1.04 ((IVSTD + LVIDD + LVPWTD) [3] – (LVIDD) [3]) + 0.6 [17, 18].

### Covariates

Gestational age at birth and birth weight were obtained from midwives and hospital registries. We obtained information on maternal age, ethnicity, educational level, folic acid use and smoking in pregnancy by questionnaire at enrolment [6]. We measured first trimester maternal blood pressure with the validated oscillometric sphygmomanometer (OMRON Healthcare Europe BV, Hoofddorp, The Netherlands).

### Statistical analyses

We examined the associations of decelerated growth and a SGA with delivery outcomes and accelerated growth after 2 years using logistic regression models. For the analyses on cardiovascular outcome measurements we constructed standard deviation score values ([observed value – mean] / SD) for the childhood cardiovascular outcome measures to enable comparison of effect estimates for the different outcomes. We did not create age adjusted standard deviation scores as the childhood outcomes were measured in a small age range and age of the child was included as a covariate in all models. We used three different linear and logistic regression models to examine the associations of decelerated growth and SGA with infant growth and childhood cardiovascular outcomes. Logistic regression was used for the outcomes decelerated growth and SGA. Childhood cardiovascular outcomes were analyzed with linear regression models. The basic model was adjusted for child’s sex, age and ethnicity. The confounder model was additionally adjusted for maternal age, maternal educational level, smoking in pregnancy and folic acid use. We selected these confounders on the basis of their associations with both the exposure and the outcome of interest and / or a change in effect estimate of more than 10%. We considered the confounder model to be the main model.

Since the group of FGR fetuses is a mixed population with both AGA and SGA fetuses, we wanted to exclude the possibility that the effect of FGR depends on being SGA. Therefore effect modification was tested on the multiplicative scale. If *p* < 0.10 was fulfilled regression analyses concerning FGR were performed in strata; SGA fetuses and AGA fetuses.

For all analyses, the percentages of missing values of covariates were lower than 20%. An overview of which covariates were used in the model and their percentage missing is presented in Additional file 1: Table S1. We imputed missing data of the covariates by using multiple imputations [19]. Ten datasets were created and analyzed together. Statistical analyses were performed using the Statistical Package of Social Sciences version 21.0 for Windows (IBM Corp., Armonk, NY, USA).

## Results

### Baseline characteristics

Baseline characteristics are shown in Table 1. SGA fetuses had a mean birth weight of 2484 ± 402 g (1.7th percentile) while fetuses with a decelerated growth had a mean birth weight of 2992 ± 383 g (21.5th percentile). Gestational age at birth was comparable between SGA fetuses and fetuses with a growth deceleration (39,9 [35,9 – 41,9] wks versus 39,7 [36,6 – 41,6] wks). Compared with growth-decelerated fetuses, the mothers of SGA fetuses were more often nulliparous, of non-Western descent and smokers.

**Table 1:** Baseline characteristics

	Total study-population	DG	SGA
	*n* = 7959	*n* = 958	*n* = 408
Maternal age	29,7 (5,2)	28,9 (5,6)	29,1 (5,7)
Anthropometrics			
Height (cm)	167,2 (7,4)	165,8 (7,1)	164,0 (7,0)
Weight (kg)	69,3 (13,3)	68,0 (13,0)	63,8 (12,5)
BMI (kg/m2)	23,8 (19,3 - 33,6)	23,7 (19,0 - 34,1)	22,7 (18,4 - 31,6)
Ethnicity			
Non-Western	3171 (41,7%)	423 (46,6%)	206 (53,4%)
Educational level			
Low	971 (12,2%)	133 (13,9%)	55 (13,5%)
Smoking habits			
Yes - continued	1482 (18,6%)	237 (24,7%)	130 (31,9%)
Folic acid use - Yes (%)			
No	2382 (29,9%)	340 (35,5%)	151 (37,0%)
Nulliparous (%)	4470 (56,2%)	610 (63,7%)	289 (70,8%)
Gestational age at birth (wks)	40,1 (37,0 - 42,0)	39,7 (36,6 - 41,6)	39,9 (35,9 - 41,9)
Preterm birth < 37 wks (%)	297 (3,7%)	37 (3,9%)	11 (2,7%)
Birthweight (g)	3412 (560)	2992 (383)	2484 (402)
Birthweight (percentile)	47,1 (28,7)	21,5 (14,8)	1,72 (1,14)
Placenta weight (gr)	620 (415–900)	555 (380–769)	480 (300–678)
Ratio placenta / birthweight	0,19 (0,04)	0,19 (0,04)	0,20 (0,04)
Pre-eclampsia (%)	157 (2,1%)	36 (4,2%)	32 (8,7%)
Sex (n, % male)	4005 (50,3%)	477 (49,8%)	222 (54,4%)

Data are represented as n (%) or as the mean (SD) or as the median (90% range)

Decelerated growth (DG) was defined as a decrease in growth of at least 40 percentiles between the second trimester and birth

Using the definition of a decrease in fetal growth of at least 40 percentiles, of all SGA fetuses 96 (23.5%) had decelerated growth. Of the AGA fetuses, 862 (11.4%) experienced decelerated growth. Of all fetuses with a decelerated growth, 90% was born AGA.

### Delivery outcomes

**Fig. 2:**
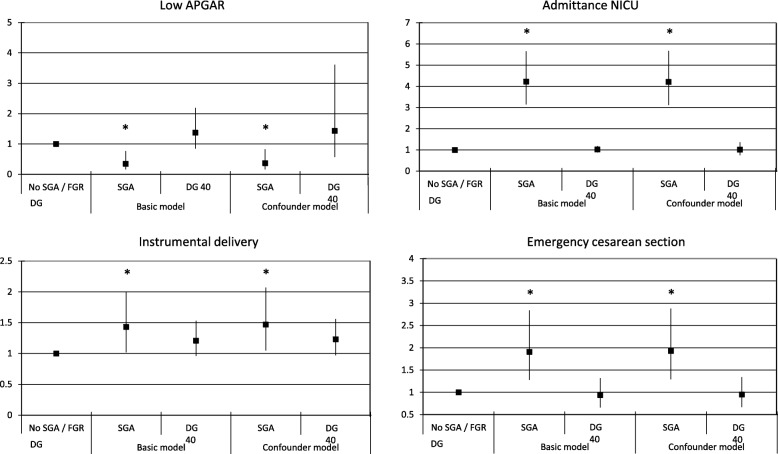
Logistic regression analyses on birth outcomes

All results on birth outcomes are presented in Fig. 2. Neonates without growth deceleration and with a normal birth weight were used as a reference category and compared with neonates with SGA or a growth deceleration of 40 percentiles (DG 40). No associations were found between decelerated growth and delivery outcomes. However, SGA was associated with a lower risk of a low APGAR score after 5 min (OR 0.37 [95% CI 0.16;0.83]) but increased risks of an instrumental delivery (OR 1.47 [95% CI 1.05;2.07]), an emergency cesarean section (OR 1.93 [95% CI 1.29;2.88]) and NICU admittance (OR 4.21 [95% CI 3.12;5.67]) compared to AGA neonates (Fig. 2)**.**

### Accelerated growth

**Fig. 3:**
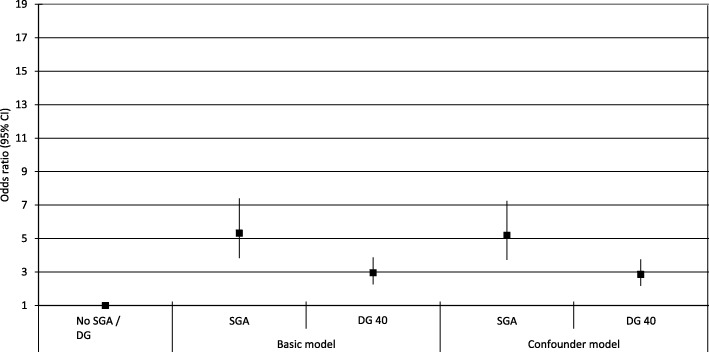
Logistic regression analyses on accelerated growth

All results on accelerated growth are presented in Fig. 3. Neonates without growth deceleration and with a normal birth weight were used as a reference category and compared with neonates with SGA or a growth deceleration of 40 percentiles (DG 40). Children born SGA had an increased risk of accelerated growth at the age of 2 years (OR 7.93 [95% CI 4.63;13.60]). However, decelerated growth was associated with an increased risk of accelerated growth at the age of 2 years (OR 2.86 [95% CI 2.17–3.76] compared to non-decelerated growth fetuses.

### Cardiovascular outcomes at the age of 6 years

All results on cardiovascular outcomes are presented in Table 2. Neonates without growth deceleration and with a normal birth weight were used as a reference category and compared with neonates with SGA, neonates with a growth deceleration (DG) and specifically neonates with a growth deceleration but a normal birth weight (DG – no SGA). Both SGA neonates as well as neonates with decelerated growth had a lower BMI (− 0.29 SD [95% CI − 0.17;− 0.41] vs (− 0.16 SD [95% CI-0.24;-0.08]), a smaller aortic root diameter (− 0.37 SD [95% CI -0.49;-0.25] vs (− 0.14 SD [95% CI-0.22;-0.06]) and lower left ventricular mass (− 0.36 SD [95% CI -0.48;-0.24] vs 0.15 SD [95% CI -0.23;-0.07]) at the age of 6 years compared to AGA neonates (Table 2).

**Table 2:** Associations between decelerated growth (DG) and cardiovascular outcomes

	Basic model		Confounder model		Childhood pathway	
	β (95% CI)	*p*-value	β (95% CI)	*p*-value	β (95% CI)	*p*-value
BMI (SD)						
No DG + no SGA	reference		reference		NA	
DG 40	-0,12 (−0,20;-0,06)	0,002	-0,16 (− 0,24;-0,08)	< 0,001		
DG 40 (AGA)	−0.09 (− 0.17;0.01)	0.01	− 0.15 (− 0.23;− 0.07)	0.001		
SGA	− 0,23 (− 0,36;− 0,12)	< 0,001	-0,29 (− 0,17;-0,41)	< 0,001		
Systolic blood pressure (SD)						
No DG + no SGA	reference		reference		reference	
DG 40	0,02 (−0,06;0,11)	0,66	-0,00 (−0,09;0,08)	0,93	0,03 (−0,06;0,11)	0,56
DG 40 (AGA)	0.01 (−0.08;0.09)	0,91	−0.01 (− 0.10;0.08)	0.85	0.04 (− 0.05;0.12)	0.71
SGA	0,12 (−0,01;0,25)	0,07	0.07 (−0.05;0.20)	0.26	0.13 (0.00;0.26)	0,04
Diastolic blood pressure (SD)						
No DG + no SGA	reference		reference		reference	
DG 40	0,07 (−0,02;0,15)	0,13	0,04 (−0,04;0,13)	0,24	0,06 (−0,03;0,14)	0,18
DG 40 (AGA)	0.06 (−0.05;0.17)	0.19	0.05 (−0.04;0.14)	0.30	0.05 (−0.04;0.14)	0.24
SGA	0.15 (0.02;0.28)	0.02	0.12 (−0.01;0.25)	0.06	0.16 (0.03;0.28)	0.02
Aortic root diameter (SD)						
No DG + no SGA	reference		reference		reference	
DG 40	−0,14 (−0,23;− 0,08)	0,001	− 0,14 (− 0,22;-0,06)	0,001	-0,10 (− 0,18;-0,03)	0,01
DG 40 (AGA)	− 0.12 (− 0.21;-0.04)	0.008	−0.12 (− 0.20;-0.03)	0.007	−0.08 (− 0.16;0.00)	0.06
SGA	−0,39 (− 0,50;− 0,27)	< 0,001	−0,37 (− 0,49;− 0,25)	< 0,001	−0,33 (− 0,44;− 0,21)	< 0,001
Left ventricular mass (SD)						
No DG + no SGA	reference		reference		reference	
DG 40	-0,17 (−0,25;-0,09)	< 0,001	-0,15 (−0,23;-0,07)	< 0,001	-0,11 (− 0,19;-0,04)	0,004
DG 40 (AGA)	−0.15 (− 0.24;-0.07)	0.001	− 0.14 (− 0.23;-0.06)	0.001	−0.09 (− 0.17;− 0.01)	0.03
SGA	−0,35 (− 0,23;− 0,47)	< 0,001	−0,36 (− 0,48;− 0,24)	< 0,001	−0,30 (− 0,41;− 0,19)	< 0,001
Pulse wave velocity (SD)						
No DG + no SGA	reference		reference		reference	
DG 40	0,00 (−0,09;0,10)	0,93	0,00 (−0,09;0,09)	0,97	-0,01 (−0,10;0,09)	0,89
DG 40 (AGA)	0.03 (−0.07;0.12)	0.57	0.03 (−0.07;0.12)	0.60	0.02 (−0.08;0.12)	0.70
SGA	-0,05 (−0,19;0,09)	0,48	-0,05 (−0,19;0,09)	0,47	-0,07 (−0,21;0,07)	0,35

Values are regression coefficient with the 95% CI and are based on linear regression models

Basic model: Adjusted child’s sex, visit interval and child’s ethnicity

Confounder model: Basic model and additionally adjusted for maternal age, educational level, smoking, folic acid intake and diastolic blood pressure at intake

Childhood pathway model: Confounder model and additionally adjusted for child’s current body mass index

*Abbreviations*: *AGA* Appropriate for gestational age birth weight, *DG 40* decelerated growth of 40 percentiles, *CI* confidence interval, *NA* not applicable

When restricting the analyses to neonates who experienced a decelerated growth with a normal birth weight (i.e. DG – AGA) the results were comparable. Results on delivery outcomes, accelerated growth and cardiovascular outcomes using the other cut-offs for defining decelerated growth are depicted in Additional file 2: Figure S1, Additional file 3: Figure S2 and Additional file 4: Figure S3 respectively.

For all outcomes no effect modification was found between decelerated growth and SGA. Our additional analyses in which we classified all fetuses with an EFW in the 2nd trimester under the 40th percentile and with a maximum decrease of growth (birth weight under the 1st percentile) as decelerated growth, showed that all the effect estimates remained the same. We concluded that potential misclassification did not affect out results. We decided to continue with the original definition i.e. a decrease of growth of at least 40 percentiles.

## Discussion

### Main findings

This study shows that both SGA as well as decelerated growth are associated with accelerated growth at the age of 2 years and altered cardiovascular measurements at the age of six. The effect estimates of the observed associations were higher for SGA than for decelerated growth. Most interestingly, substantial associations were found between fetuses with a decelerated growth but a normal birth weight, and accelerated growth at the age of 2 years and cardiovascular outcomes at the age of 6 years.

### Strengths and limitations

The main strength of this study was the extensive prospective data collection on fetal growth, childhood health and environmental influences. This enabled us to adjust for multiple confounding factors and investigate the effects of fetal growth on three different time points (i.e. at delivery and at the age of two and 6 years) in a large sample of 7959 participants.

Follow-up data at 6 years was available in 65% of our study population. Those who were not included in the study were more frequently lower educated, had a higher prevalence of multiparity, smoked more often and used less often folic acid during pregnancy (Additional file 5**:** Table S2). A more healthy population was therefore included in our study which might have introduced a selection bias and may affect the generalizability of our results.

Estimation of fetal weight with ultrasound and the Hadlock formula has a mean absolute error of 8–13% dependent of the size of the fetus [20]. However, there is a risk of overestimation in pregnancies with suspected large for gestational age (LGA) fetuses and an underestimation in pregnancies with suspected SGA [21]. Since the definition of decelerated growth used in this study is based on the percentiles of EFW this may have led to misclassification. In that case the associations between decelerated growth and our outcomes would have been biased towards the null. Hence, our associations might reflect underestimations. Moreover deviations in growth could also result from random measurement errors of the ultrasound technique. Therefore we decided to not explore a decrease in growth of less than 30 percentiles with the disadvantage that it is challenging to classify a fetus as a growth decelerated fetus if the EFW in the second trimester is p40 or below.

Since we assessed EFW in the second trimester of pregnancy a possible growth deceleration before the 20th week of gestation was not assessed and therefore missed. In the case of early growth deceleration with a normal growth in the second and third trimester of pregnancy this could have led to misclassification. A fetus would wrongfully be assigned to the group without deceleration, which would underestimate our results.

### Interpretation

One of the key findings of this study is that neonates with a decelerating growth curve have in an increased risk of accelerated growth and altered cardiovascular outcomes at the age of 6 years despite the fact that 90% of decelerated growth neonates are born AGA. In this study, we defined decelerated growth independent of birth weight or other measurements during pregnancy such as a fetal abdominal circumference under the 5th percentile, the pulsatility index of the umbilical or the middle cerebral artery or biomarkers. Since there is no consensus on how much a growth curve needs to deviate before it can be designated as a deviating growth curve several cut-offs were used based on a decrease in growth expressed in percentiles. A disadvantage of this approach is that the change in weight per percentile is not constant but increases towards the more sparsely populated extremes of a distribution. Hence, a fetus initially at the 90th percentile of a weight distribution and ending at the 50th percentile is, expressed in estimated fetal weight, more growth decelerated compared with a fetus initially at the 70th percentile and ending at the 30th percentile. Only a portion of the neonates born small for gestational age had decelerated growth, ranging from 15.9 to 32.8% depending on the used cut-off. The fact that the group of SGA fetuses is a heterogeneous group consisting out of fetuses that are constitutionally small, and fetuses with a growth deceleration is well known and accepted. In previous research on SGA different attempts were made in trying to stratify these two groups by calculating the ponderal index, use the birth weight of a sibling as a reference, or the usage of customized charts or prediction models [22–28]. However, in all these studies attention was solely focused on SGA neonates in whom decelerated growth is merely a form of SGA. Neonates born AGA are not subject of investigation whereas this study shows that 88.9 to 90.7% of the neonates with a growth deceleration were born AGA and that from all AGA born fetuses 6.9 to 17.4% is in fact growth decelerated. Despite these high percentages substantial associations between decelerated growth and accelerated growth and altered cardiovascular measurements during childhood were found. Already during pregnancy fetuses with a decelerating growth curve have a higher pulsatility index of the umbilical artery compared with fetuses without a growth restriction (*p* < 0.01). A sensitivity analyses was performed repeating the analysis in only growth-decelerated fetuses born AGA, which gave the same results (data not shown). This indicates that growth decelerated neonates born AGA should be considered as a high risk group with more emphasize needed in future research.

The effect estimates of the associations on accelerated growth at the age of two and cardiovascular measurements at the age of six were higher for SGA children compared with those with growth deceleration. One might hypothesize that this implies that birth weight, the endpoint of a growth pattern, is more important than the growth pattern itself. However, it could also be that the effects are not measurable yet in case the fetus did not reach a certain lower limit of birth weight (i.e. a threshold effect). The difference in birth weight between SGA neonates and neonates with a growth deceleration could explain why associations were found with delivery outcomes for SGA neonates but not for growth decelerated neonates. SGA neonates were more often delivered by an emergency cesarean section or an instrumental delivery compared with growth-decelerated neonates. Due to their low birth weight SGA neonates are more prone to experience fetal distress with as consequence an increased risk of seizures, respiratory diseases, hypoglycemia and hyperbilirubinemia with admittance at the NICU compared with their AGA counterparts [29]. This foresight could have influenced the practicing physician by lowering the threshold when to perform an emergency cesarean section. This would also explain why preterm birth before 37 weeks of gestation occurs more often in SGA fetuses compared with growth decelerated fetuses (3.9% vs 2.7%, Table 1). Partly this will be iatrogenic due to the knowledge that the fetus is SGA. This tendency to intervene earlier might be an explanation why SGA neonates more often are delivered by vaginal instrumental delivery or cesarean section, in the presence but perhaps also absence of non-reassuring fetal heart rate monitoring, but less often have an APGAR score below seven after 5 min. This is not the case for FGR fetuses since the majority of these fetuses were born AGA. However, in the neonatal model in which we additionally adjusted for birth weight the effect on emergency cesarean section and admittance at the NICU department remains significant, meaning that other factors besides birth weight are of importance.

Both growth deceleration as well as SGA was associated with an increased risk of accelerated growth in the first 2 years of life. Accelerated growth is associated with obesity in later life giving rise to impaired cardiovascular health [30]. Especially in those born with a low birth weight [31, 32]. This is in line with our study in which the SGA neonates, who had a higher risk of accelerated growth compared with the growth decelerated neonates, also had poorer cardiovascular outcomes. One could also explain the associations between SGA and cardiovascular outcomes by body size since children born SGA often have a lower BMI compared with their peers.

Growth deceleration is not limited to mid and late pregnancy only. Embryonic growth and development during the first trimester of pregnancy is essential for organogenesis of the fetal cardiovascular system. Impaired early growth has also been shown to be associated with an adverse cardiovascular risk profile in children at the age of 6 years [33, 34]. If fetal growth restriction occurs in early pregnancy, gestational age is often adjusted according to the crown-rum-length. After adjustment fetal growth may seem appropriate although the neonate should have been classified as being SGA with the long-term sequalae as shown in this article. Therefore more attention is needed for fetal growth restriction throughout gestation and not only during the second half of pregnancy.

Not only SGA neonates but also growth decelerated neonates with a normal birth weight had a different cardiovascular profile at the age of 6 years as shown by differences in the aortic root diameter and left ventricular mass. It is well established that low birth weight is associated with poor cardiovascular health in later life but the fact that also growth pattern is a risk factor independent of birth weight is an important finding. A smaller aortic root diameter is associated with ventricular outflow obstruction and possibly the development of hypertension in later life. While it is different to relate these measurement to exact cardiac function in later adult life, our findings strongly suggest that growth decelerated neonates despite their normal birth weight already have a less optimal cardiovascular profile which warrants follow-up and further investigation.

## Conclusion

In this study in which we explored differences in outcome between SGA and growth decelerated fetuses, we observed that fetuses with a decelerated growth curve but nevertheless are born AGA constitute a vulnerable group with increased risks of accelerated growth and altered cardiovascular outcomes in childhood and in their future lives. Future research should be focused on this particular group since these newborns are now, focusing on only a birthweight < p10, not classified as such and are not subject to any follow up in future life.

## Additional files

Additional file 1: **Table S1.** Multiple Imputations Model. (DOCX 14 kb)

Additional file 2: **Figure S1.** Associations between fetal growth restriction and delivery outcomes. (PDF 334 kb)

Additional file 3: **Figure S2.** Associations between fetal growth restriction and accelerated growth. (PDF 255 kb)

Additional file 4: **Figure S3.** Associations between fetal growth restriction and cardiovascular outcomes. (PDF 476 kb)

Additional file 5: **Table S2.** Baseline characteristics between included and excluded participants at the age of 6 years. (DOCX 17 kb)

## Data Availability

The datasets used and/or analyzed during the current study are available from the corresponding author on reasonable request.

## Abbreviations

AC: Abdominal circumference
AGA: Appropriate for gestational age
AOD: Aortic root diameter
BMI: Body mass index
DBP: Diastolic blood pressure
DG: Decelerated growth
DOHaD: Development and Origins of Health and Disease theory
EFW: Estimated fetal weight
FGR: Fetal growth restriction
FL: Femur length
HC: Head circumference
IVSTD: Intra-ventricular septum thickness in diastole
LGA: Large for gestational age
LVIDD: Left ventricular internal diameter in diastole
LVM: Left ventricular mass
LVPWTD: Left ventricular posterior wall thickness in diastole
MEC: Medical Ethics Committee
NICU: Neonatal intensive care unit
PWV: Pulse wave velocity
SBP: Systolic blood pressure
SD: Standard deviation
SGA: Small for gestational age
