# Supplementary material for: Deceleration of fetal growth rate as alternative predictor for childhood outcomes: a birth cohort study
Source: BMC Pregnancy Childbirth. 2019 Jun 27;19:216. doi: 10.1186/s12884-019-2358-8 (PMC6598289; doi:10.1186/s12884-019-2358-8)
Supplement: Supplementary file 1 — Table S1. Multiple Imputations Model. (DOCX 14 kb) [file 12884_2019_2358_MOESM1_ESM.docx]

Additional file: Table S1 Multiple Imputations Model

| Covariate | Percentage missing |
| --- | --- |
| Maternal ethnicity | 4,5% |
| Maternal educational level | 0% |
| Smoking behavior | 11,4% |
| Folic acid intake | 1,4% |
| Maternal diastolic blood pressure (1^st^ trimester) | 3,8% |
| BMI of the child at age 6 | 1,2% |
| Gestational age at birth | 0% |
| Parity | 0,2% |
| Maternal BMI at intake | 3,6% |
| Breastfeeding | 8,9% |
| Maternal height | 3,2% |
| Fetal growth (SGA and/or DG) | 0% |

For the imputation model the Markov Chain Monte Carlo method was used. Ten datasets were created and analysed together.
