# Supplementary material for: Deceleration of fetal growth rate as alternative predictor for childhood outcomes: a birth cohort study
Source: BMC Pregnancy Childbirth. 2019 Jun 27;19:216. doi: 10.1186/s12884-019-2358-8 (PMC6598289; doi:10.1186/s12884-019-2358-8)
Supplement: Supplementary file 2 — Figure S1. Associations between fetal growth restriction and delivery outcomes. (PDF 334 kb) [file 12884_2019_2358_MOESM2_ESM.pdf]

Additional file 2: Figure S1 Associations between fetal growth restriction and delivery outcomes

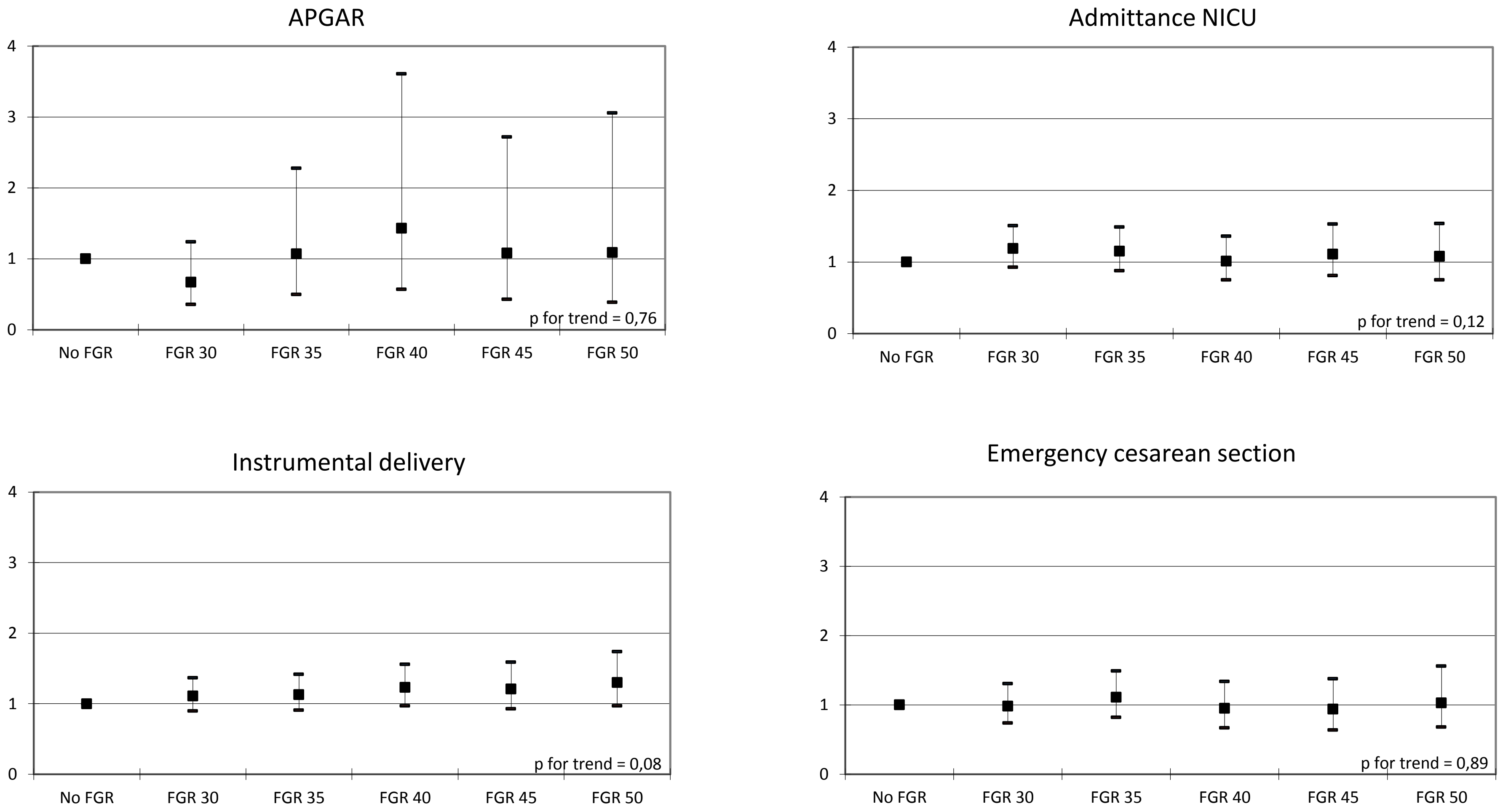

Values represent odds ratios with the 95% confidence interval of the confounder model that reflects the difference in delivery outcomes between fetal growth restriction (FGR) as compared to the reference group (no FGR). Models were adjusted for child's age, sex, ethnicity, maternal age, educational level, smoking, folic acid intake and diastolic blood pressure at intake. Trend lines are only given when p-value for linear trend < 0,05. NICU = neonatal intensive care unit.
