# Supplementary material for: Deceleration of fetal growth rate as alternative predictor for childhood outcomes: a birth cohort study
Source: BMC Pregnancy Childbirth. 2019 Jun 27;19:216. doi: 10.1186/s12884-019-2358-8 (PMC6598289; doi:10.1186/s12884-019-2358-8)
Supplement: Supplementary file 3 — Figure S2. Associations between fetal growth restriction and accelerated growth. (PDF 255 kb) [file 12884_2019_2358_MOESM3_ESM.pdf]

Aditional file 3: Figure S2 Associations between fetal growth restriction and accelerated growth

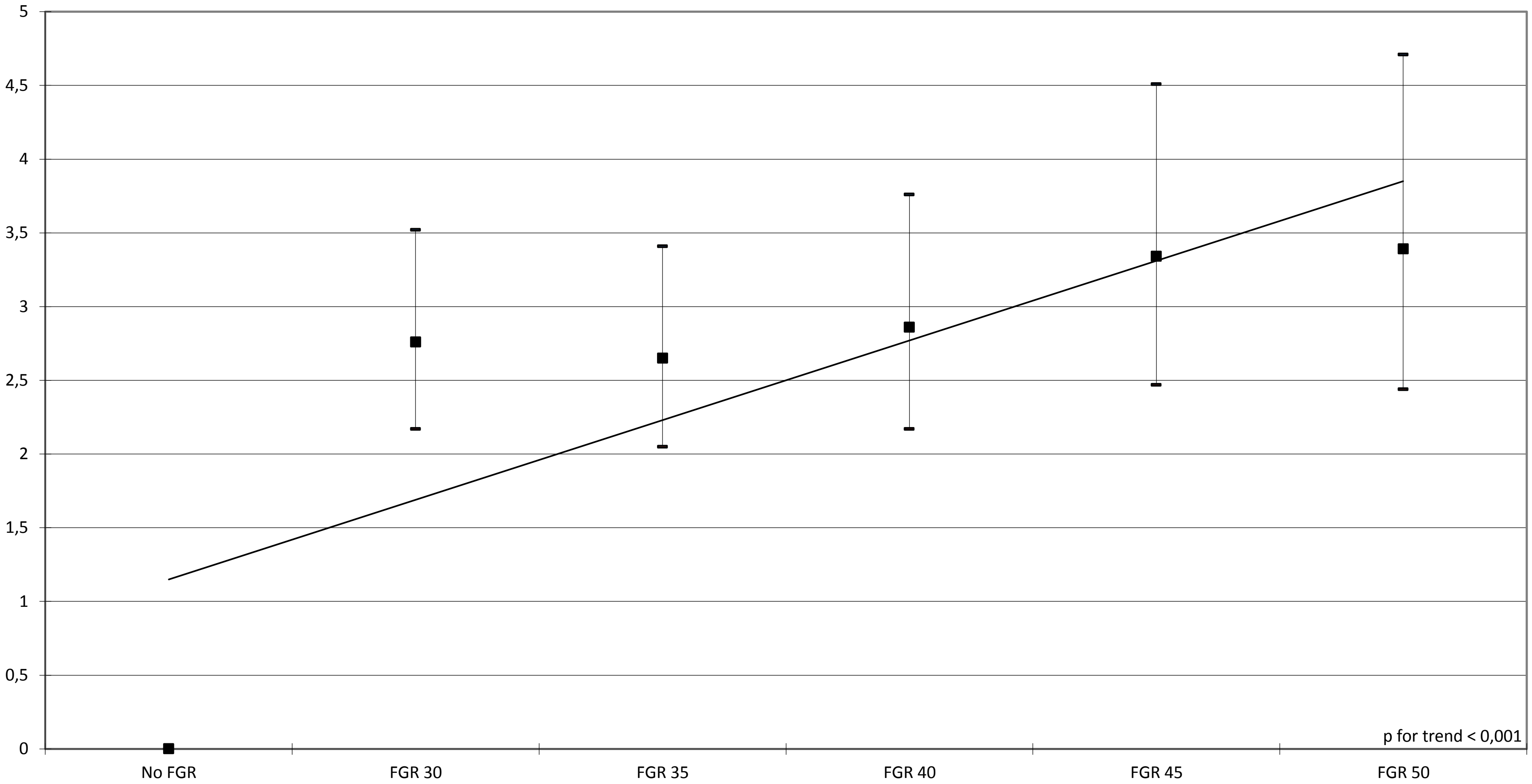

Values represent odds ratios with the 95% confidence interval of the confounder model that reflects the difference in accelerated growth between fetal growth restriction (FGR) as compared to the reference group (no FGR). Models were adjusted for child's age, sex, ethnicity, maternal age, educational level, smoking, folic acid intake and diastolic blood pressure at intake. Trend lines are only given when p-value for linear trend < 0,05.
