# Supplementary material for: Deceleration of fetal growth rate as alternative predictor for childhood outcomes: a birth cohort study
Source: BMC Pregnancy Childbirth. 2019 Jun 27;19:216. doi: 10.1186/s12884-019-2358-8 (PMC6598289; doi:10.1186/s12884-019-2358-8)
Supplement: Supplementary file 4 — Figure S3. Associations between fetal growth restriction and cardiovascular outcomes. (PDF 476 kb) [file 12884_2019_2358_MOESM4_ESM.pdf]

Additional file 4: Figure S3 Associations between fetal growth restriction and cardiovascular outcomes

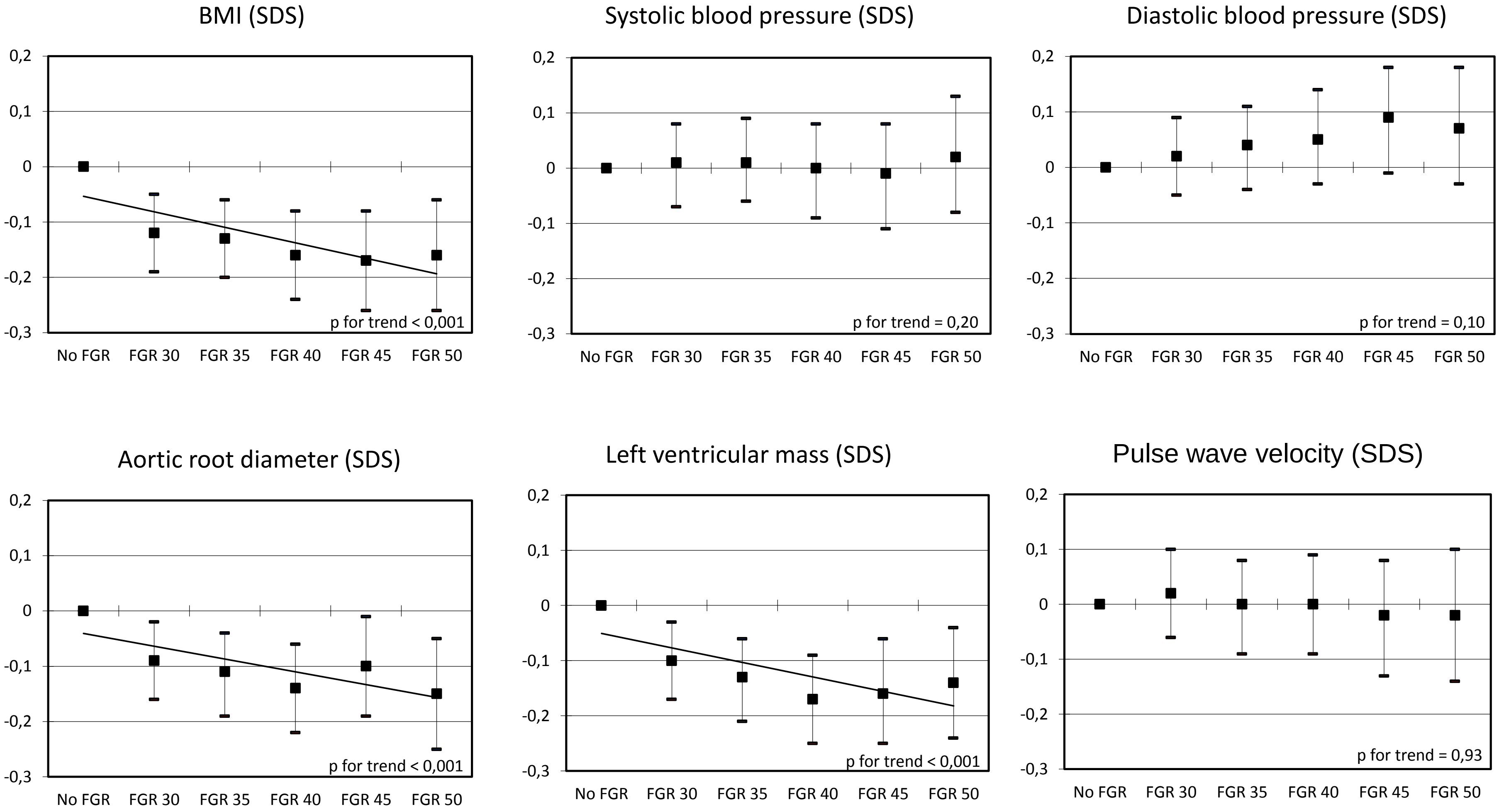

Values represent linear regression coefficients with the 95% confidence interval of the confounder model that reflect the difference in childhood outcomes expressed as standard deviation socres (SDS) between fetal growth restriction (FGR) as compared to the reference group (no FGR). Models were adjusted for child's age, sex, ethnicity, maternal age, educational level, smoking, folic acid intake and diastolic blood pressure at intake. Trend lines are only given when p-value for linear trend < 0,05.
