# Supplementary material for: Deceleration of fetal growth rate as alternative predictor for childhood outcomes: a birth cohort study
Source: BMC Pregnancy Childbirth. 2019 Jun 27;19:216. doi: 10.1186/s12884-019-2358-8 (PMC6598289; doi:10.1186/s12884-019-2358-8)
Supplement: Supplementary file 5 — Table S2. Baseline characteristics between included and excluded participants at the age of 6 years. (DOCX 17 kb) [file 12884_2019_2358_MOESM5_ESM.docx]

| **Additional file: Table S2 Baseline characteristics between included and excluded participants at the age of 6 years** | | | |
| --- | --- | --- | --- |
|  |  |  |  |
|  |  | Included | Excluded |
|  |  | n = 5173 | n = 2786 |
| **Maternal age** | | 29,7 (5,2) | 29.9 (5,1) |
| **Anthropometrics** | |  |  |
|  | Height (cm) | 167,2 (7,4) | 167,1 (7,4) |
|  | Weight (kg) | 69,3 (13,3) | 69,8 (13.4) |
|  | BMI (kg/m2) | 23,8 (19,3 - 33,6) | 24,1 (18,4 – 33,7) |
| **Ethnicity** | |  |  |
|  | Non-Western | 3171 (41,7%) | 1303 (46,8%) |
| **Educational level** | |  |  |
|  | Low | 971 (12,2%) | 610 (21,9%) |
| **Smoking habits** | |  |  |
|  | Yes - continued | 1482 (18,6%) | 652 (23,4%) |
| **Folic acid use - Yes (%)** | |  |  |
|  | No | 2382 (29,9%) | 629 (22,6%) |
| **Nulliparous (%)** | | 4470 (56,2%) | 1348 (48,4%) |
| **Gestational age at birth (wks)** | | 40,1 (37,0 - 42,0) | 40,0 (37,0 – 42,0) |
| **Preterm birth <37 wks (%)** | | 297 (3,7%) | 109 (3,9%) |
| **Birthweight (g)** | | 3412 (560) | 3386 (558) |
| **Birthweight (percentile)** | | 47,1 (28,7) | 46,6 (28,6) |
| **Placenta weight (gr)** | | 620 (415 - 900) | 614 (410 – 880) |
| **Ratio placenta / birthweight** | | 0,19 (0,04) | 0,19 (0,05) |
| **Pre-eclampsia (%)** | | 157 (2,1%) | 61 (2,2%) |
| **Sex (n, % male)** | | 4005 (50,3%) | 1407 (50,5%) |
|  |  |  |  |
